# BMC *Pregnancy and Childbirth* reviewer acknowledgement 2014

**DOI:** 10.1186/s12884-015-0443-1

**Published:** 2015-02-18

**Authors:** Nawsheen Boodhun

**Affiliations:** BioMed Central, Floor 6, 236 Gray’s Inn Road, London, WC1X 8HB UK

## Abstract

The editors of BMC Pregnancy and Childbirth would like to thank all our reviewers who have contributed to the journal in Volume 14 (2014).

**Vigdis Aasheim**

Norway

**Edgardo Abalos**

Argentina

**Parvin Abedi**

Iran

**Peter Abrahams**

UK

**Barbara Abrams**

USA

**Ishag Adam**

Sudan

**Babatunde Adedokun**

Nigeria

**Mulat Adefris**

Ethiopia

**Ramesh Adhikari**

Nepal

**Asri Adisasmita**

Indonesia

**Werner Adler**

Germany

**Philip Adongo**

Ghana

**Abdallah Adra**

Lebanon

**Dinesh Agarwal**

India

**Mukesh Mansha Agarwal**

United Arab Emirates

**Schadrac Agbla**

Benin

**Kingsley Agho**

Australia

**Priya Agrawal**

USA

**Regina Aguiar**

Brazil

**Charles Agyemang**

Netherlands

**Jamil Ahmed**

Pakistan

**Saifuddin Ahmed**

USA

**Noori Akhtar-Danesh**

Canada

**Stella Akinleye**

Nigeria

**Olufunke Alaba**

South Africa

**Ashraful (Neeloy) Alam**

Australia

**Salvatore Alberico**

Italy

**Juan Luis Alcazar**

Spain

**Sophie Alexander**

Belgium

**Charles Algert**

Australia

**Victoria Allen**

Canada

**Nedal Alnawaiseh**

Jordan

**Charles Ameh**

UK

**Richard Amenyah**

Burkina Faso

**Amanda Ampt**

Australia

**Sarah Amugongo**

Kenya

**Judith Anchang-Kimbi**

Cameroon

**Anne-Marie Nybo Andersen**

Denmark

**Kathryn Andersen**

USA

**Cynthie Anderson**

USA

**Nasratullah Ansari**

Afghanistan

**Marcela Agustina Araya Bannout**

Chile

**Brigid Arkins**

Ireland

**Raul Artal**

USA

**Atnafu Getachew Asfaw**

Ethiopia

**Catherine Ashwin**

UK

**Nega Assefa**

Ethiopia

**Lou Atkinson**

UK

**Laura Attanasio**

USA

**Ingvild Aune**

Norway

**Marie-Paule Austin**

Australia

**Francisco Avila**

Chile

**Jennifer Ayton**

Australia

**Hassan Ba'Aqeel**

Saudi Arabia

**Frank Baarveld**

Netherlands

**Rashmi Bagga**

India

**Patricia Bailey**

USA

**Lesley Baillie**

USA

**Emily Bain**

Australia

**Kathleen Baird**

Australia

**Murat Bakacak**

Turkey

**Marian Bakker**

Netherlands

**Wouter Bakker**

Netherlands

**Marie-Clare Balaam**

UK

**Tatiana Balachova**

USA

**Subha Sri Balakrishnan**

India

**Julius Bamidele**

Nigeria

**Germana Bancone**

Thailand

**Sunita Bandewar**

Canada

**Melanie Bannister-Tyrrell**

Australia

**Flora Maria Barbosa Da Silva**

Brazil

**Lesley Barclay**

Australia

**Madeleine Barnett**

UK

**Mauro Barni**

Italy

**Hyam Bashour**

Syria

**Sriparna Basu**

India

**B Bateman**

USA

**Linda Bauld**

UK

**Hamideh Bayrampour**

Canada

**Eva Bazant**

USA

**Sarah Beake**

UK

**Roxanne Beauclair**

South Africa

**Bérengère Beauquier-Maccotta**

France

**Helen Bedford**

UK

**Carol Bedwell**

UK

**Katrien Beeckman**

Belgium

**Andrea Begley**

Australia

**Yaakov Beilin**

USA

**Mesfin Awoke Bekalu**

Ethiopia

**Mireille Bekker**

Netherlands

**Mandy Bell**

USA

**Katrien Benhalima**

Belgium

**Karen Benzies**

Canada

**Marie Berg**

Sweden

**Annette Bernloehr**

Germany

**Peter Berti**

Canada

**Miteshkumar Bhanderi**

India

**Divya Kanwar Bhati**

India

**Sohinee Bhattacharya**

UK

**Yagya Bhurtyal**

UK

**Debra Bick**

UK

**Cara Bicking Kinsey**

USA

**Lorena Binfa**

Chile

**Connie Bish**

USA

**Antje Blank**

Germany

**Hannah Blencowe**

UK

**Ellen Blix**

Norway

**Paula Bolton-Maggs**

UK

**Jermane Bond**

USA

**Marie-Pierre Bonnet**

France

**Elizabeth Bonney**

USA

**Matteo Bonomo**

Italy

**Matteo Bonzini**

Italy

**Matthias Borchert**

Germany

**Georgios Bouras**

Greece

**Marielle Karine Bouyou-Akotet**

Gabon

**Angela Bowen**

Canada

**Carol Bower**

Australia

**Katherine Bowers**

USA

**Elaine Boyle**

UK

**Marek Brabec**

Czech Republic

**Loretta Brabin**

UK

**Carmel Bradshaw**

Ireland

**Petra Brhlikova**

UK

**Annette Briley**

UK

**Carinne Brody**

USA

**Sharon Bruce**

Canada

**Sherri Bucher**

USA

**Eckhart Buchmann**

South Africa

**Henna Budhwani**

USA

**Irina Buhimschi**

USA

**Anne Buist**

Australia

**Maria Bullarbo**

Sweden

**Joanna Busza**

UK

**Elizabeth Butrick**

USA

**Alexander Butwick**

USA

**Abbey Byrne**

Australia

**Joanne Cacciatore**

USA

**Jennifer Callaghan-Koru**

USA

**Wendy Camelo Castillo**

USA

**Christina Campbell**

USA

**Angela Carberry**

Australia

**Michael Carson**

USA

**John Carstensen**

Sweden

**Christine Catling-Paull**

Australia

**Ricardo Cavalli**

Brazil

**Juraci Cesar**

Brazil

**Dong Hyun Cha**

Korea South

**Christina Chambers**

USA

**Ronna Chan**

USA

**Yi-Sheng Chao**

Canada

**Lucy Chappell**

UK

**Lisa Chasan-Taber**

USA

**Sarika Chaturvedi**

India

**Picklu Chaudhuri**

India

**Phaikyeong Cheah**

Thailand

**Yingyao Chen**

China

**Li-Yin Chien**

Taiwan

**Karmel Choi**

USA

**Abdul Mannan Choudhury**

Bangladesh

**Ole Bjarne Christiansen**

Denmark

**Cynthia Chuang**

USA

**John Cleland**

UK

**Tim Colbourn**

UK

**John Condon**

Australia

**Christine Connors**

Australia

**Stephen Contag**

USA

**Martina Cornel**

Netherlands

**Giuliana Cortese**

Italy

**Maria Laura Costa**

Brazil

**Karen Cowgill**

USA

**Denis Crankshaw**

Canada

**Debra Creedy**

Australia

**Jenny Cresswell**

UK

**David Cromwell**

UK

**Doug Cronie**

Netherlands

**Rhonda Curran**

UK

**Andrew E. Czeizel**

Hungary

**Fabricio Da Silva Costa**

Australia

**Per Damkier**

Denmark

**Cem Dane**

Turkey

**Francesco D'Antonio**

Italy

**Zoe Darwin**

UK

**Sandra David Tchouda**

France

**Michael Davies**

Australia

**Deborah Davis**

Australia

**Angela Dawson**

Australia

**Vincent De Brouwere**

Belgium

**Gaia De Campora**

Italy

**Johanna De Graaf**

Netherlands

**Claire De Labrusse**

Switzerland

**Francine De Montigny**

Canada

**Paul De Reu**

Netherlands

**Eugene Declercq**

USA

**Tedbabe Degefie**

Ethiopia

**Stephanie Dellicour**

UK

**Stephanie Delong**

USA

**Anna Dencker**

Sweden

**Barbara Dennison**

USA

**Costanzo Di Maria**

UK

**Mirko Di Martino**

Italy

**Nadia Diamond-Smith**

USA

**Cyril Dim**

Norway

**Cyril Dim**

Nigeria

**Jodie Dionne-Odom**

USA

**Khady Diouf**

USA

**Mai Do**

USA

**Jodie Dodd**

Australia

**Edel Doherty**

Ireland

**Sara Donahue**

USA

**Amol Dongre**

India

**Soo Downe**

UK

**Michele Drehmer**

Brazil

**Mari Dumbaugh**

Switzerland

**Corinne Dupont**

France

**Jill Durocher**

USA

**Elizabeth Duthie**

USA

**Indranil Dutta**

India

**Johannes Duvekot**

Netherlands

**Alexandra Duxbury**

UK

**Els Duysburgh**

Belgium

**Pratibha Dwarkanath**

India

**Michelle Dynes**

USA

**Christine East**

Australia

**Thomas Ebert**

Germany

**Winifred Eboh**

UK

**Elizabeth Echoka**

Kenya

**Michael Ediau**

Uganda

**Roger Edwards**

USA

**Bolaji Egbewale**

Nigeria

**Anne Ego**

France

**Samantha Ehrlich**

USA

**Kristjana Einarsdottir**

Australia

**Maria Ekelin**

Sweden

**Alison El Ayadi**

USA

**Julio Elito Jr.**

Brazil

**Waleed El-Khayat**

Egypt

**Sohier Elneil**

UK

**Susheela Engelbrecht**

USA

**Cyril Engmann**

USA

**Offer Erez**

Israel

**Jan Jaap H.M. Erwich**

Netherlands

**Olapeju Esimai**

Nigeria

**Michael Esplin**

USA

**Birgitta Essén**

Sweden

**Holly Essex**

UK

**Titilayo Fakeye**

Nigeria

**Shangrong Fan**

China

**Sara Farchi**

Italy

**Marcelo Farias**

Chile

**Bukola Fawole**

Nigeria

**Maya Fehling**

Germany

**Marlena Fejzo**

USA

**Gedefaw Abeje Fekadu**

Ethiopia

**Tanis Fenton**

Canada

**Jennifer Fenwick**

Australia

**Leonides Fernandez**

Spain

**Enrico Ferrazzi**

Italy

**Catherine Fetherston**

Australia

**Tamara Fetters**

USA

**Francesc Figueras**

Spain

**Kirstin Finning**

UK

**Jane Fisher**

Australia

**Mirembe Florenc**

Uganda

**Andrew Fogarty**

UK

**Bruno M Fonseca**

Portugal

**Jane Ford**

Australia

**Liz Forty**

UK

**Jean Christophe Fotso**

Kenya

**Maralyn Foureur**

Australia

**Mirjam Fransen**

Netherlands

**Jennifer Fraser**

Australia

**Heather Frey**

USA

**Yechiel Friedlander**

Israel

**J. Frederik Froen**

Norway

**Suthat Fucharoen**

Thailand

**Judith Fullerton**

USA

**Anita Gagnon**

Canada

**Jenny Gamble**

Australia

**Rebecca Ganann**

Canada

**Robin Gandley**

USA

**Marie Gantz**

USA

**Ana Garces**

Guatemala

**Deepak Garg**

UK

**Susan Garthus-Niegel**

Norway

**Deirdre Gartland**

Australia

**Asheber Gaym**

Ethiopia

**Abebaw Gebeyehu**

Ethiopia

**Yirgu Gebrehiwot**

Ethiopia

**Alem Gebremariam**

Ethiopia

**Samson Gebremedhin**

Ethiopia

**Diederike Geelhoed**

Mozambique

**Ado Geidam**

Nigeria

**Caitlin Gerdts**

USA

**Rebecca Giallo**

Australia

**Frances Gibson**

Australia

**Kelly Gibson**

USA

**Melanie Gibson-Helm**

Australia

**Emma L Giles**

UK

**Katie Gillies**

UK

**Erik Giltay**

Netherlands

**Sarah Gimbel**

USA

**Yehuda Ginosar**

Israel

**Beatriz F. Giraldo**

Spain

**Fiseha Girma**

Ethiopia

**Carmen Giurgescu**

USA

**Salvatore Gizzo**

Italy

**Svetlana Glinianaia**

UK

**Marewa Patricia Glover**

New Zealand

**Katherine Goetzinger**

USA

**Srinivas Goli**

India

**Gustavo Gonzales**

Peru

**Adrienne Gordon**

Australia

**Michael Gravett**

USA

**Richard Griffey**

USA

**Malcolm Griffiths**

UK

**Peta Grigsby**

USA

**Rosalie Grivell**

Australia

**Karin Gross**

Switzerland

**Pietro Grussu**

Italy

**Susanne Grylka-Baeschlin**

Germany

**Ruth Guinsburg**

Brazil

**Marie-Julia Guittier**

Switzerland

**Anil Gumber**

UK

**Freedom Nkhululeko Gumedze**

South Africa

**Ricardo Gurgel**

Brazil

**Ipek Gurol-Urganci**

UK

**Muhammad Atif Habib**

Australia

**Dereje Habte**

Botswana

**Eleni Hadjigeorgiou**

Cyprus

**Jeannie Haggerty**

Canada

**Demewoz Haile**

Ethiopia

**Desta Hailu**

Ethiopia

**Arja Halkoaho**

Finland

**Wendy Hall**

Canada

**Stephen Halpern**

Canada

**Mohga Hammad**

Egypt

**Karin Hammarberg**

Australia

**Gillian Hanley**

Canada

**Tharangrut Hanprasertpong**

Thailand

**Claudia Hanson**

Sweden

**Jessica Hanson**

USA

**Philip Harber**

USA

**Susanne Harder**

Denmark

**Lorie Harper**

USA

**Junichi Hasegawa**

Japan

**Mahmoud Hassan**

France

**Hossein Hassani**

UK

**Elizabeth Hatch**

USA

**Abigail Hatcher**

South Africa

**Marie Hatem**

Canada

**Margaretha Haugen**

Norway

**T Lee-Ann Hawkins**

Canada

**Louise Hayes**

UK

**Maureen Heaman**

Canada

**Alexander Heazell**

UK

**Steffie Heemelaar**

Netherlands

**Rebecca Helmreich**

USA

**Jane Henderson**

UK

**Jennifer Henderson**

Australia

**Amanda Henry**

Australia

**Edgar Hernandez Andrade**

USA

**Florian Herse**

Germany

**Nicola Heslehurst**

UK

**Philip Hess**

USA

**Jenny Hewison**

UK

**Briony Hill**

Australia

**Zelee Hill**

UK

**Lisa Hinton**

UK

**Janet Hirst**

UK

**Abraham Hodgson**

Ghana

**Hans V Hogerzeil**

Netherlands

**Anna-Clara Hollander**

Sweden

**Jennifer Hollowell**

UK

**Sara Holton**

Australia

**Caroline Homer**

Australia

**Claire Homeyard**

UK

**Jaco Homsy**

USA

**Dell Horey**

Australia

**Shigeko Horiuchi**

Japan

**Gerard Hornstra**

Netherlands

**Jaime Horton**

USA

**John Horwood**

New Zealand

**Nazli Hossain**

Pakistan

**Sennen Hounton**

Burkina Faso

**Wendy Hoy**

Australia

**Amber Hromi-Fiedler**

USA

**Daniel Hruschka**

USA

**Kun Huang**

China

**Carl Hubel**

USA

**Megan Huchko**

USA

**Thomas Hulsey**

USA

**Tracy Humphrey**

UK

**Tai-Ho Hung**

Taiwan

**Jon Hyett**

Australia

**Lawrence Ikamari**

Kenya

**Jane Iles**

UK

**Lawrence Impey**

UK

**Victor Inem**

Nigeria

**Bilal Iqbal Avan**

UK

**Por Ir**

Cambodia

**Chibuike Iruloh**

UK

**M Mazharul Islam**

Oman

**Aditi Iyer**

India

**Nicola Jackson**

Thailand

**Laurie James-Hawkins**

USA

**Elisabeth Jangsten**

Sweden

**Patricia Janssen**

Canada

**Pouya Javadian**

USA

**Larissa Jennings**

USA

**Esther Jimenez**

Spain

**Helene Johns**

Australia

**Kate Jolly**

UK

**Julie Jomeen**

UK

**Catriona Jones**

UK

**Sasi Jonnalagadda**

USA

**Ruby Jose**

India

**K Joseph**

Canada

**Chandni Joshi**

Australia

**Racape Judith**

Belgium

**Othman Kakaire**

Uganda

**Douglas Kalman**

USA

**Christabel Kambala**

Germany

**Ahmed Kamel Eldesouky**

Egypt

**Martin Kammerer**

UK

**Lucy Kanya**

Kenya

**Marianna Karachaliou**

Greece

**Rajendra Karkee**

Nepal

**Fyson Kasenga**

Malawi

**Noriko Kato**

Japan

**Bekana Kebede**

Ethiopia

**William Keenan**

USA

**Susan Kelly**

UK

**Shona Kelly**

UK

**Bryn Kemp**

UK

**Tamil Kendall**

USA

**Holly Kennedy**

USA

**Sara Kenyon**

UK

**Rosemary Keogh**

Australia

**Michail Keramidas**

Sweden

**Asma Khalil**

UK

**Monir Khalil**

Libya

**Attia Khan**

Pakistan

**Michelle Khan**

USA

**Shane Khan**

USA

**Vishnu Khanal**

Nepal

**Soghra Khani**

Iran

**Sunil Khanna**

USA

**Ali S Khashan**

Ireland

**Sharon Kibwana**

Ethiopia

**Hussein Lesio Kidanto**

Tanzania

**Masahiro Kihara**

Japan

**Deborah Kim**

USA

**Jo Kim**

USA

**Jinseok Kim**

Korea South

**Elizabeth Kimani-Murage**

Kenya

**Carol Kingdon**

UK

**Dawn Kingston**

Canada

**Brooke Kinniburgh**

Canada

**Tarja I Kinnunen**

Finland

**Russell Kirby**

USA

**Pertti Kirkinen**

Finland

**Sharon Kirkpatrick**

Canada

**Dmitry Kissin**

USA

**Kristen Kjerulff**

USA

**Michael Klein**

Canada

**Atle Klovning**

Norway

**Hannah Knight**

UK

**Marian Knight**

UK

**Thecla Kohi**

Tanzania

**Theano Kokkinaki**

Greece

**Owonikoko Kola**

Nigeria

**Klaas Koop**

Netherlands

**Tomomi Kotani**

Japan

**Mirjana Kovac**

Serbia

**Katy Kozhimannil**

USA

**Naoko Kozuki**

USA

**Karoline Kragelund Nielsen**

Denmark

**Gv Krishnaveni**

India

**Carsten Krüger**

Germany

**Andrzej Kulczycki**

USA

**Lily C Kumbani**

Malawi

**Su-Chen Kuo**

Taiwan

**Zehra Kurdoglu**

Turkey

**Juan Kusanovic**

USA

**Raman Kutty**

India

**Anne Stine Kvehaugen**

Norway

**Linda Kvist**

Sweden

**Harm Jan Kwikkel**

Netherlands

**Elisabeth Kylberg**

Sweden

**Catherine Kyobutungi**

Kenya

**Elisa Lacerda**

Brazil

**Eve Lackritz**

USA

**Ching Tat Lai**

Australia

**Hugh Simon Hung San Lam**

Hong Kong

**Steven Lamm**

USA

**Jens Langhoff-Roos**

Denmark

**Sophia Lannon**

USA

**Hermann Lanou**

Burkina Faso

**Annunziata Lapolla**

Italy

**Martha Lappas**

Australia

**Elysia Larson**

USA

**Anne-Marie Laslett**

Australia

**Laura Lauria**

Italy

**Tina Lavender**

UK

**Denise Lawler**

Ireland

**Beverley Lawton**

New Zealand

**Liana Leach**

Australia

**Nicky Leap**

Australia

**Meng-Chih Lee**

Taiwan

**Sue Lee**

Thailand

**Ana Leslie**

Brazil

**Felicia Lester**

USA

**Ky Leung**

Hong Kong

**Adrienne Levay**

Canada

**Sarah Lewis**

UK

**Changwei Li**

USA

**Ching-Chiang Lin**

Taiwan

**Helena Lindgren**

Sweden

**Robert Liston**

Canada

**Siu-Wai Lit**

Hong Kong

**James Litch**

USA

**Ching-Ming Liu**

Taiwan

**Tsz Kin Lo**

China

**Robert Locke**

USA

**Terhi Lohela**

Finland

**Elin Bjørge Løken**

Norway

**Carl Lombard**

South Africa

**Mary Longworth**

UK

**Yolanda Lopez**

Spain

**Jorge Lopez-Camelo**

Argentina

**Fred Lotgering**

Netherlands

**Jimmy Chun Yu Louie**

Australia

**Svetla Loukanova**

Germany

**Mona Loutfy**

Canada

**Julia Lowe**

Canada

**Richard Lowe**

USA

**Miha Lucovnik**

Slovenia

**Mirjam Lukasse**

Norway

**Pisake Lumbiganon**

Thailand

**Ingela Lundgren**

Sweden

**Karsten Lunze**

USA

**Zhong-Cheng Luo**

Canada

**Angela Lupattelli**

Norway

**Jennifer Lutomski**

Ireland

**Courtney Lynch**

USA

**Nanna Maaløe**

Denmark

**Karen Mackinnon**

Canada

**Mohsen Maddah**

Iran

**Moke Magoma**

Tanzania

**Michael Johnson Mahande**

Tanzania

**Qun Mai**

Australia

**Abdulkarim Mairiga**

Nigeria

**Emma Louise Malchau Carlsen**

Denmark

**Maili Malin**

Finland

**Caroline Maltepe**

Canada

**Albert Manasyan**

Zambia

**Justin Mandala**

USA

**Jeevan Marasinghe**

Sri Lanka

**Dannette Marie**

UK

**Lena B Martensson**

Sweden

**Helene Martin**

Switzerland

**Rocio Martin**

Netherlands

**Wellington Martins**

Brazil

**Samantha Mascelli**

Italy

**Gileard Masenga**

Tanzania

**Edwin Massey**

UK

**Matthews Mathai**

Switzerland

**Ilan Matok**

Israel

**Hiroshi Matsushita**

Japan

**Anne Matthews**

Ireland

**Robyn Maude**

New Zealand

**Godfrey Michael Mbaruku**

Tanzania

**Columba K Mbekenga**

Tanzania

**Judith Mcara-Couper**

New Zealand

**Emma Mccall**

UK

**Janya Mccalman**

Australia

**Elizabeth Mccarthy**

Australia

**Anthony Desmond Mccarthy**

Argentina

**Elizabeth Mcclure**

USA

**Colin Mccord**

UK

**Christine Mccourt**

UK

**Ellie Mcdonald**

Australia

**Susan Mcdonald**

Australia

**Ted Mcdonald**

Canada

**Alison Mcfadden**

UK

**Rose Mcgready**

Thailand

**Tania Mcintosh**

UK

**Meredith Mcintyre**

Australia

**Helen Mclachlan**

Australia

**Tarek Meguid**

Malawi

**Tarek Meguid**

Namibia

**Azar Mehrabadi**

Canada

**Yared Mekonnen**

Ethiopia

**N Melamed**

Israel

**Antonella Mencacci**

Italy

**Sotero Mengue**

Brazil

**Ramkumar Menon**

USA

**Fiona Mensah**

UK

**Astrid Merkx**

Netherlands

**Abi Merriel**

UK

**Amy Metcalfe**

Canada

**Carla Meurk**

Australia

**Jill Mhyre**

USA

**Antonina Mikocka-Walus**

UK

**Annie Mills**

Australia

**Ghazala Mir**

UK

**Jezid Miranda**

USA

**Shiva Raj Mishra**

Nepal

**Malgorzata Miszkurka**

Canada

**Bryan Mitchell**

Canada

**Prasanna Mithra**

India

**Ankita Mittal**

India

**Blandina Theophil Mmbaga**

Norway

**D Paul Moberg**

USA

**Vernon Mochache**

Kenya

**Ingrid Mogren**

Sweden

**Ben Mol**

Afghanistan

**Mitike Molla**

Ethiopia

**Mark Monahann**

Ireland

**Rob Mooij**

Netherlands

**Tiffany Moore Simas**

USA

**Tahereh Moradi**

Sweden

**Imran Morhason-Bello**

Nigeria

**Rafael Moroni**

Brazil

**Pamela Morrison**

UK

**Michelle Mottola**

Canada

**Cheryl Moyer**

USA

**Mwifadhi Mrisho**

Tanzania

**Projestine Muganyizi**

Tanzania

**Ashar Muhammad Malik**

Pakistan

**Peter Mukasa**

Uganda

**Kathryn Muldoon**

Ireland

**Luke Mullany**

USA

**Sunni Mumford**

USA

**Zubia Mumtaz**

Canada

**Sylvia Murphy Tighe**

Ireland

**Andrew James Murray**

UK

**Jonah Musa**

Nigeria

**Adamson Muula**

Malawi

**Beatrice Mwagomba**

Malawi

**Ipyana Mwampagatwa**

Tanzania

**Emanuel Mwendo**

Tanzania

**Elizabeth Nabiwemba**

Uganda

**Harriet Nabudere**

Uganda

**Mona Nabulsi**

Lebanon

**Manisha Nair**

UK

**Hacer Nalbant**

Turkey

**Jane Namasasu**

Malawi

**Bejoy Nambiar**

Malawi

**Kavita Nanda**

USA

**Jolly Nankunda**

Uganda

**Rahel Nardos**

USA

**Natasha Nassar**

Australia

**Dipty Nawal**

India

**Juliet Ndibazza**

Uganda

**Ellen Nelissen**

Tanzania

**Sutapa Neogi**

India

**Gustavo Neppelenbroek**

Brazil

**Robin Nesbitt**

Germany

**John Newell**

Ireland

**James Newham**

UK

**Shu-Kay Angus Ng**

Australia

**Caroline Nguluwe**

Malawi

**Thinh Nguyen**

Australia

**Susan Niermeyer**

USA

**Marianne Nieuwenhuijze**

Netherlands

**Bahareh Nikooyeh**

Iran

**Christina Nilsson**

Sweden

**Somashekhar Nimbalkar**

India

**I Nippert**

Germany

**Tanya Nippita**

Australia

**Yoshihiro Nishida**

Japan

**Kaijun Niu**

Japan

**Elie Nkwabong**

Cameroon

**Roseli M Y Nomura**

Brazil

**Randa Nooh**

Saudi Arabia

**Shane Norris**

South Africa

**Angelo Nyamtema**

Tanzania

**Kerstin H Nyqvist**

Sweden

**Anicet Nzabonimpa**

Rwanda

**Jeremy Oats**

Australia

**Colm Oboyle**

Ireland

**Beverley O'Brien**

Canada

**Pat O'Brien**

UK

**Michel Odent**

UK

**Ju Lee Oei**

Australia

**Olanma Ogbuehi**

UK

**Kerry-Ann O'Grady**

Australia

**Tinuade Ogunlesi**

Nigeria

**Magdalena Ohaja**

Ireland

**Felix Okah**

USA

**Okechukwu Angelis Okezie**

Botswana

**Ellinor Olander**

UK

**Oladapo Olayemi**

Nigeria

**Joann O'Leary**

USA

**Purevdorj Olkhanud**

USA

**Per Olofsson**

Sweden

**Christine Olson**

USA

**David Olson**

Canada

**Bolajoko O. Olusanya**

Nigeria

**Ayodeji Oluwole**

Nigeria

**Siti Zawiah Omar**

Malaysia

**Sinead M. O'Neill**

Ireland

**Maricianah Atieno Onono**

Kenya

**Sharad Onta**

Nepal

**Marieke Oostvogels**

Netherlands

**Carlos Ortega González**

Mexico

**Alfred Osoti**

Kenya

**David Osrin**

UK

**Eugene Oteng-Ntim**

UK

**Ellis Owusu-Dabo**

Ghana

**Semra Ozdemir**

Singapore

**Carmen Pace**

Australia

**Julie Pallant**

Australia

**Chandrakant Pandav**

India

**Sarita Panday**

Nepal

**Damaru Paneru**

Nepal

**Eleni Papadopoulou**

Norway

**Theodore Papaioannou**

Greece

**Shantini Paranjothy**

UK

**Min Hae Park**

UK

**Angela Pascall**

UK

**Dharmintra Pasupathy**

UK

**Jillian Patterson**

Australia

**Deepak Paudel**

Nepal

**Deborah Payne**

New Zealand

**Christopher Pell**

Netherlands

**Andrea Barnabas Pembe**

Tanzania

**Suzanne Penfold**

UK

**Suzanne Penfold**

Slovakia

**Teuila Percival**

New Zealand

**Gloria Pérez**

Spain

**Susan Perlen**

Australia

**Amber Peterman**

USA

**Wendy Peterson**

Canada

**Stamatios Petousis**

Greece

**Constanze Pfeiffer**

Switzerland

**Sharon Phelan**

USA

**Suparat Phuanukoonnon**

Papua New Guinea

**Maria Piancino**

Italy

**Jashvant Poeran**

Netherlands

**Paras K Pokharel**

Nepal

**Wendy Pollock**

Australia

**Nancy Poole**

Canada

**Maree Porter**

Australia

**Malcolm Potts**

USA

**Jennifer Powers**

Australia

**Robert Powers**

USA

**Ndola Prata**

USA

**Holly Priddis**

Australia

**Danielle Kaye Prime**

Australia

**Samantha Prosser**

Australia

**Audrey Prost**

UK

**Wendy Prudhomme O'Meara**

Kenya

**Roopa Ps**

India

**Giuseppe Puccio**

Italy

**Caroline Pukall**

Canada

**Eero Pukkala**

Finland

**Shuby Puthussery**

UK

**Xu Qian**

China

**Liqian Qiu**

China

**Parvin Rahnama**

Iran

**Sari Räisänen**

Finland

**Augustine Rajakumar**

USA

**Tonse Raju**

USA

**Meenakshi Ramphul**

Ireland

**Heribert Ramroth**

Germany

**Deborah Randall**

Australia

**Bharat Randive**

India

**Svein Rasmussen**

Norway

**Erica Rauff**

USA

**Anita Ravelli**

Netherlands

**Joanna Raven**

UK

**Sundari Ravindran**

India

**Joel Ray**

Canada

**Angela Reichelt**

Brazil

**Joerg Reichert**

Germany

**Cavan Reilly**

USA

**Liv Merete Reinar**

Norway

**Kristina Martha Renault**

Denmark

**Udo Reulbach**

Ireland

**Annette Reuss**

Germany

**Grazzia Rey**

Uruguay

**Richard Rheingans**

USA

**Kalisa Richard**

Rwanda

**Carol Richardson**

UK

**Anne Rifkin-Graboi**

Singapore

**Machteld Rijken**

Netherlands

**Marcus Rijken**

Thailand

**Marcus Rijken**

Netherlands

**Marlies Rijnders**

Netherlands

**Monica Rittler**

Argentina

**Demetrios Rizos**

Greece

**Julie Robitaille**

Canada

**Dianne Rodger**

Australia

**Anna Joy Rogers**

USA

**Cheong Rae Roh**

Korea South

**Liliam Cristine Rolo Paiato**

Brazil

**Ted Rosales**

Canada

**Alan Rothberg**

South Africa

**Ingrid Rowlands**

Australia

**Jenny Ruducha**

USA

**Stephen Rulisa**

Rwanda

**Alice Rumbold**

Australia

**Audrey Saftlas**

USA

**Joanne Said**

Australia

**Evelyn Sakeah**

Ghana

**Kabiru Salami**

Nigeria

**Sarah Saleem**

Pakistan

**Raed Salim**

Israel

**Jenny Salmon**

New Zealand

**Birgitta Salomonsson**

Sweden

**Jacob Sandberg**

Sweden

**Julia Sanders**

UK

**Eduardo Santana**

Brazil

**Nejc Sarabon**

Slovenia

**Carolyn Sargent**

USA

**Narendra Nath Sarkar**

India

**Benn Sartorius**

South Africa

**Sadath Sayeed**

USA

**Jules Schagen Van Leeuwen**

Netherlands

**Ashley Schempf**

USA

**Virginia Schmied**

Australia

**Georg Schmolzer**

Canada

**Christiane Schwarz**

Germany

**Jane Scott**

Australia

**Marie Scully**

UK

**Anna Seale**

UK

**Hannah Motshedisi Sebitloane**

South Africa

**Kerem Doga Seckin**

Turkey

**Innocent Semali**

Tanzania

**Tetine Sentell**

USA

**Karen Sepucha**

USA

**Marie Seraphin**

USA

**Elisabeth Severinsson**

Norway

**Prakeshkumar Shah**

Canada

**Antonia Shand**

Australia

**Caitlin Shannon**

USA

**Sheetal Sharma**

UK

**Sudesh Raj Sharma**

Nepal

**Alison Shaw**

UK

**Zoe Sheppard**

UK

**Ashalatha Shetty**

UK

**Eiji Shibata**

Japan

**Shashikant Sholapurkar**

UK

**Binjwala Shrestha**

Nepal

**Alexis Shub**

Australia

**Joseph Shumway**

USA

**Crystal Silva**

USA

**Robert Silver**

USA

**Kyra Sim**

Australia

**Leickness Chisamu Simbayi**

South Africa

**Melissa Simon**

USA

**Nigel Simpson**

UK

**Gunjan Singh**

United Arab Emirates

**Kavita Singh**

USA

**Rameet Singh**

USA

**Sodiomon Bienvenu Sirima**

Burkina Faso

**Deborah Sitrin**

USA

**Valeria Skafida**

UK

**Nancy L Sloan**

USA

**Maria Small**

USA

**Rhonda Small**

Australia

**Debbie Smith**

UK

**Jeffrey Michael Smith**

USA

**Julie Smith**

Australia

**Valerie Smith**

Ireland

**John Smulian**

USA

**Erna Snelgrove-Clarke**

Canada

**Jonathan Snowden**

USA

**Malin Soederberg**

Sweden

**Andrea Solnes Miltenburg**

Netherlands

**Priya Soma-Pillay**

South Africa

**Ingvil Krarup Sørbye**

Norway

**Alexandros Sotiriadis**

UK

**Carina Sparud-Lundin**

Sweden

**Georgina Spies**

South Africa

**Henri Spronk**

Netherlands

**Chandrashekhar Sreeramareddy**

Nepal

**Anke Steckelberg**

Germany

**M Steinhoff**

USA

**Jelle Stekelenburg**

Netherlands

**Olof Stephansson**

Sweden

**Alexandre Stephens**

Australia

**Joanna Stewart**

New Zealand

**Mary Stewart**

UK

**Zoe Stewart**

UK

**Sarah Stock**

UK

**Kathrin Stoll**

Germany

**Hege Therese Størksen**

Norway

**Zhixian Sui**

Australia

**Sa'Adatu Sule**

Nigeria

**Bo Sun**

China

**Johanne Sundby**

Norway

**Fernanda G C Surita**

Brazil

**Elisabeth Svensson**

Sweden

**Elizabeth Taglauer**

Ireland

**Mizuki Takegata**

Japan

**Yin On Yvonne Tam**

USA

**Berhanu Tameru**

USA

**Fangbiao Tao**

China

**Belaynew Taye**

Ethiopia

**Lee Taylor**

Australia

**Kate Teela**

USA

**Gerben Ter Riet**

Netherlands

**Christian Teter**

USA

**Li Thies-Lagergren**

Sweden

**Gill Thomson**

UK

**James Tielsch**

USA

**Christiana Titaley**

Indonesia

**Deirdre Tobias**

USA

**Rachel Tolhurst**

UK

**Gabriele Tonni**

Italy

**Jocelyn Toohill**

Australia

**Maria Regina Torloni**

Brazil

**Christopher Torrens**

UK

**Siranda Torvaldsen**

Australia

**Clare Tower**

UK

**Thach Tran**

Viet Nam

**Naomi Trengove**

Australia

**Dominic Trepel**

UK

**Vandana Tripathi**

USA

**Amy Tsui**

USA

**Sandy Tubeuf**

UK

**Ozge Tuncalp**

USA

**Deborah Turnbull**

Australia

**Nana A. Y. Twum-Danso**

USA

**Zia Ul-Haq**

UK

**Odi' Ugochukwu Joannes Umeora**

Nigeria

**Aniefiok Umoiyoho**

Nigeria

**Julia Unterscheider**

Australia

**Michele Upvall**

USA

**Michael Urban**

South Africa

**Julio Urrutia**

Chile

**Ihab Usta**

Lebanon

**Michele Usuelli**

Italy

**Bettina Utz**

Belgium

**Marja Vääräsmäki**

Finland

**Edi Vaisbuch**

USA

**Christine Valente**

UK

**Frédérique Vallières**

Ireland

**Jodie Valpied**

Australia

**Heleen Van Beekhuizen**

Netherlands

**Rob Van Dam**

Singapore

**Peter Van De Ven**

Netherlands

**Jef Van Den Ende**

Belgium

**Albertine Van Der Does**

South Africa

**Frits Van Der Haar**

USA

**Jacoba Van Der Kooy**

Netherlands

**Carla Van Der Wijden**

Netherlands

**Nickie Van Der Wulp**

Netherlands

**Paul Van Kesteren**

Netherlands

**An-Sofie Van Parys**

Belgium

**Lenie Van Rossem**

Netherlands

**Vicki Van Wagner**

Canada

**Christiaan Van Woerden**

Netherlands

**Jeroen Vanderhoeven**

USA

**Linda Vanotoo**

Ghana

**Paula Vargas Innocenti**

Chile

**Manu Vatish**

UK

**Saraswathi Vedam**

Canada

**Katri Vehvilainen-Julkunen**

Finland

**Marjolein Verhart**

Netherlands

**Stefan Verlohren**

Germany

**Tienke Vermeiden**

Ethiopia

**Rose Victor**

Tanzania

**Francesco Vierucci**

Italy

**Jenny Vieveen**

Netherlands

**Simone Vigod**

Canada

**Anne Britt Vika Nilsen**

Norway

**Divya Vohra**

USA

**Frauke Von Versen-Höynck**

Germany

**Franziska Wadephul**

UK

**Yohannes Dibaba Wado**

Ethiopia

**Lorraine Walker**

USA

**Dilys Walker**

USA

**L. Lewis Wall**

USA

**Julia Walsh**

USA

**Yuping Wang**

USA

**Carol Wanjira**

Kenya

**Rhoda Wanyenze**

Uganda

**Nicola Wardrop**

UK

**Lucie Warren**

UK

**Arjumand Warsy**

Saudi Arabia

**Lyndsey Watson**

Australia

**Andrew Weeks**

UK

**Mary Nell Wegner**

USA

**Carolyn Weiniger**

Israel

**Diana Wellesley**

UK

**Barbara Welles-Nystrom**

USA

**Michael Wells**

Sweden

**Shi Wu Wen**

Canada

**Heather Whitford**

UK

**Eva Wiberg-Itzel**

Sweden

**Therese Wiegers**

Netherlands

**Hanne Benedicte Wielandt**

Denmark

**Ingela Wiklund**

Sweden

**Carol Wilkins**

UK

**Chris Wilkinson**

Australia

**Shelley Wilkinson**

Australia

**Jane Willcox**

Australia

**Calistus Wilunda**

Italy

**Martha Wingate**

USA

**Brita Askeland Winje**

Norway

**Katherine Wisner**

USA

**Cynthia Wong**

USA

**Cindy Woods**

Australia

**Abebaw Worku**

Ethiopia

**Francesca Wuytack**

Ireland

**Karen Wynter**

Australia

**Haipeng Xiao**

China

**Xu Xiong**

USA

**Mohammad Yakoob**

USA

**Mohsin Yakub**

USA

**Eileen Yam**

USA

**Gavin Yamey**

USA

**Junta Yanai**

Japan

**Zhenyu Yang**

China

**Hung-Wen Yeh**

USA

**Jane Yelland**

Australia

**Tegbar Yigzaw**

Ethiopia

**Susanha Yimyam**

Thailand

**Koyo Yoshida**

Japan

**David Young**

Canada

**Sera Young**

USA

**Shamsa Zafar**

Pakistan

**Partamin Zainullah**

Afghanistan

**Vincenzo Zanardo**

Italy

**Giovanni Zanconato**

Italy

**Phyllis Zelkowitz**

Canada

**Lubo Zhang**

USA

**Jing Zheng**

USA

**Mariusz Zimmer**

Poland

**Marcelo Zubaran Goldani**

Brazil

**Luisa Zuccolo**

UK

**Rose Zulliger**

USA

**Prisca Ac Zwanikken**

Netherlands

